# Current Challenges in the Post-Transplant Care of Liver Transplant Recipients in Germany

**DOI:** 10.3390/jcm9113570

**Published:** 2020-11-05

**Authors:** Kerstin Herzer, Martina Sterneck, Martin-Walter Welker, Silvio Nadalin, Gabriele Kirchner, Felix Braun, Christina Malessa, Adam Herber, Johann Pratschke, Karl Heinz Weiss, Elmar Jaeckel, Frank Tacke

**Affiliations:** 1 Department of Gastroenterology and Hepatology, University Hospital Essen, University Duisburg-Essen, 45147 Essen, Germany; kerstin.herzer@kbs.de; 2 Knappschafts-Klinik Bad Neuenahr, 53474 Bad Neuenahr-Ahrweiler, Germany; 3 Department of Medicine, University Medical Center Hamburg Eppendorf, 20251 Hamburg, Germany; sterneck@uke.de; 4 Department of Internal Medicine I, University Hospital Frankfurt, 60590 Frankfurt am Main, Germany; Martin-Walter.Welker@kgu.de; 5 Department for General, Visceral and Transplant Surgery, University Hospital Tuebingen, 72016 Tuebingen, Germany; Silvio.Nadalin@med.uni-tuebingen.de; 6 Department of Surgery, University Hospital of Regensburg, 93053 Regensburg, Germany; Gabriele.Kirchner@klinik.uni-regensburg.de; 7 Innere Medizin I, Caritaskrankenhaus St. Josef, 93053 Regensburg, Germany; 8 Department for Transplantation Surgery, University Hospital Kiel, 24105 Kiel, Germany; Felix.Braun@uksh.de; 9 Department of General, Visceral and Vascular Surgery, University Hospital Jena, 07747 Jena, Germany; Christina.Malessa@med.uni-jena.de; 10 Department of Gastroenterology and Rheumatology, University Hospital Leipzig, 04103 Leipzig, Germany; Adam.Herber@medizin.uni-leipzig.de; 11 Department of Surgery, Campus Charité Mitte/Campus Virchow-Klinikum, Charité University Medicine Berlin, 13353 Berlin, Germany; johann.pratschke@charite.de; 12 Berlin Institute of Health, 13353 Berlin, Germany; 13 Department of Internal Medicine, University of Heidelberg, 69120 Heidelberg, Germany; KarlHeinz.Weiss@med.uni-heidelberg.de; 14 Department of Internal Medicine, Salem Medical Center, 69120 Heidelberg, Germany; 15 Integrated Research and Treatment Centre Transplantation (IFB-Tx), Hannover Medical School, 30625 Hannover, Germany; Jaeckel.Elmar@mh-hannover.de; 16 Department of Gastroenterology, Hepatology and Endocrinology, Hannover Medical School, 30625 Hannover, Germany; 17 Department of Hepatology & Gastroenterology, Campus Charité Mitte/Campus Virchow-Klinikum, Charité University Medicine Berlin, 13353 Berlin, Germany

**Keywords:** liver transplantation, long-term outcome, post-liver transplantation management, liver transplantation center, aftercare structures, Germany

## Abstract

Improving long-term patient and graft survival after liver transplantation (LT) remains a major challenge. Compared to the early phase after LT, long-term morbidity and mortality of the recipients not only depends on complications immediately related to the graft function, infections, or rejection, but also on medical factors such as de novo malignancies, metabolic disorders (e.g., new-onset diabetes, osteoporosis), psychiatric conditions (e.g., anxiety, depression), renal failure, and cardiovascular diseases. While a comprehensive post-transplant care at the LT center and the connected regional networks may improve outcome, there is currently no generally accepted standard to the post-transplant management of LT recipients in Germany. We therefore described the structure and standards of post-LT care by conducting a survey at 12 German LT centers including transplant hepatologists and surgeons. Aftercare structures and form of cost reimbursement considerably varied between LT centers across Germany. Further discussions and studies are required to define optimal structure and content of post-LT care systems, aiming at improving the long-term outcomes of LT recipients.

## 1. Introduction

Liver transplantation (LT) remains the treatment of choice for selected patients with end-stage liver disease or acute liver failure [1,2,3]. While early post-LT 1-year survival rates have essentially improved to over 80% [4], this does not seem to apply equally to the long-term outcome. The 10-year patient survival data obtained from two different time periods (1990–1994 and 2005–2007) did not improve, with 60% and 61%, respectively [5]. In fact, long-term survivors are at continued risk of increased morbidity [2,5]. In addition to “classical” transplant-related complications such as graft dysfunction, rejection, or liver disease recurrence, the late mortality is also due to factors that are independent of the graft function. Metabolic complications, cardiovascular disease, renal dysfunction, and extrahepatic malignancies play a major role in the long-term morbidity and mortality of liver transplant patients. Thus, the long-term post-LT management is complex and requires a close follow-up and multidisciplinary approach to recognize, manage, and prevent medical complications and comorbidities [5,6].

To the best of our knowledge, there is no generally accepted nationwide standard to the post-LT management either in Germany or in other countries. Up until now, it has also not sufficiently evaluated as to when and which patients will benefit from which aftercare structures, follow-up procedures, or surveillance strategies. A first German guideline on liver transplantation is currently being prepared and is expected to be published in 2021. To gain a first insight into variations in the aftercare structures and delivery of post-transplant care in Germany, a group of hepatologists and surgeons met and discussed current coordination and practice patterns of outpatient care after LT in Germany. On the basis of survey data collected in this sample of German transplant units and the view of the expert panel, we have highlighted the main topics in the post-transplant care of LT recipients in Germany below: medical challenges that affect the post-LT long-term outcome, challenges of optimizing immunosuppressive medication, and specific challenges in structural landscape of LT aftercare within the LT centers and between the sectors.

### 1.1. Medical Challenges in the Post-Transplant Care after LT

#### 1.1.1. Metabolic Complications after LT

Metabolic syndrome and related disorders are an increasing challenge in the management of LT recipients contributing to late post-operative morbidity and mortality [1]. According to the most common definition, the presence of any three of five risk factors (elevated fasting glucose, reduced High-density lipoprotein (HDL) cholesterol, elevated triglycerides, obesity, or hypertension) constitutes a diagnosis of metabolic syndrome (International Diabetes Federation [IDF]/American Heart Association [AHA]/National Heart, Lung, and Blood Institute [NHLBI], Alberti et al., 2009). The metabolic syndrome can on the one hand be the cause of a chronic liver disease and on the other hand contribute to the progression of a liver disease of another cause [5]. In fact, many LT recipients have a present or past history of metabolic syndrome and at-risk cardiovascular (CV) profiles with a high prevalence of hypertension, hyperlipidemia, and recurrent diabetes mellitus (DM) or new-onset diabetes mellitus after transplantation (NODAT) [2,5].

Despite the expected increasing relevance of CV diseases among LT candidates, there have only been a few studies to date that have dealt with CV risk factors at the time of LT or the predisposing factors for the development of a future CV disease in LT recipients. In a multicenter study evaluating the prevalence and evolution of CV risk factors and CV-related morbidity/mortality over a 5 year period after LT (*n* = 1819), most of the CV events were observed within the first year after LT, suggesting that pre-existing conditions play a major role in determining the CV-associated morbidity and mortality of LT recipients [7]. In a cohort study of LT recipients (*n* = 994) on the incidence of major adverse cardiac events (MACE), sustained transient post-transplant diabetes (PTDM) was significantly associated with increased long-term risk of MACE (13% and 27% after 5 and 10 years, respectively) [7].

Similar to the United States, where the number of adults with non-alcoholic steatohepatitis (NASH) awaiting LTs has almost tripled between 2004 and 2013, and in light of the expected increase in people with overweight (body mass index [BMI] 25–30 kg/m^2^) and obesity (BMI > 30 kg/m^2^), it can be assumed that non-alcoholic fatty liver disease (NAFLD) will develop to a major cause of end-stage liver disease in Germany [8,9]. NAFLD is already the most common liver disease in Germany, and the number of patients with NASH-related cirrhosis is projected to increase by more than 100% in the next decade [10]. The post-transplant period due to either relapse or de novo development of NAFLD is also gaining importance. In most NASH patients, steatosis seems to recur after LT, although fibrosis was shown not to progress as rapidly in the later post-transplant years (4–5 years) as it does before LT [11]. In a large prevalence study (*n* = 548), significant steatosis (grade 2 and 3) was associated with a trend toward increased CV mortality [12].

However, specific recommendations regarding prevention or treatment of NAFLD or NASH in LT recipients have not been established thus far. The international practice guidelines for “liver transplantation” and “long-term management of the successful adult liver transplant” recommend avoiding excessive gain in body weight and optimizing management of hypertension and diabetes (both in pre- and post-transplant settings) [1,2]. Similarly, the current German guidelines for NAFLD management recommend a regular check-up of the CV and metabolic risk profiles as well as normalization of body weight and optimization of DM treatment [13]. Although the evidence to support one immunosuppressive regimen over another in patients who undergo LT for NASH/cirrhosis is limited, the United States and German guidelines are in favor of minimizing corticosteroids [2,13]. The American Association for the Study of Liver Diseases (AASLD) and the American Society of Transplantation (AST) recommend to screen patients for DM (fasting plasma glucose every 3 months in the first year and then annually, review of self-monitoring of blood glucose every 3 months and HbA1c-monitoring every 3 months with intervention at glycosylated hemoglobin [HbA1c] levels ≥ 7.0%). Additionally, an annual screening on diabetic complications (e.g., retinopathy), microalbuminuria, and dyslipidaemia is recommended in new-onset or pretransplant DM (PDM) [2]. The European Association for the Study of the Liver (EASL) mentions the need for a “continuous cardiovascular risk stratification” and the “rapid detection and treatment of metabolic disorders, as well as modification of risk factors”, including tailoring the immunosuppressive regimen [1]. Among LT recipients who become severely or morbidly obese and fail behavioral weight-loss programs, the AASLD recommends considering bariatric surgery, even if the optimal procedure and timing still need to be clarified [2].

#### 1.1.2. Post-Transplant Renal Dysfunction after LT

Chronic renal dysfunction is recognized as a very common complication after transplantation of nonrenal organs. An analysis of a longitudinal U.S. database of 36,389 LT recipients transplanted between 1990 and 2000 indicated that the prevalence of kidney failure (defined as glomerular filtration rate of 29 mL/minute/1.73 m^2^ of body surface area or less or the development of end-stage renal disease (ESRD)) was 18% at 5 years and 25% at 10 years [2,14]. For patients with renal insufficiency, factors significantly associated with worse survival included higher age at the time of transplantation, higher creatinine, PDM, and transplantation in the pre-MELD (model of end-stage liver disease) era [15]. However, as the MELD score is driven in part by serum creatinine as a marker of renal function, implementation of the MELD scoring system shifted donor liver prioritization to transplant candidates with renal dysfunction [16]. Possible causes of impaired kidney function after LT include (long-term) exposure to immunosuppressive regimes based on calcineurin inhibitors (CNI), preoperative kidney dysfunction, perioperative acute kidney injury/hypertension, DM, and atherosclerosis pre- and/or post-LT [1].

To prevent post-transplant chronic kidney disease (CKD), continuous monitoring of renal function in LT recipients, adjustment of the immunosuppression (usually on an individual level, especially in patients with impaired kidney function), and the control of DM and hypertension as well as avoidance of nephrotoxic drugs are recommended [1,5]. The AASLD and the AST recommend that the glomerular filtration rate and the proteinuria are assessed at least once a year [2].

#### 1.1.3. De Novo Malignancies after LT

De novo malignancies are also one of the leading causes of death beyond 1 year post-LT [17], and in terms of the future, they are expected to become the major cause of late mortality in LT recipients [18]. Several observational studies have reported that, depending on the duration of follow-up, the risk of solid de novo neoplasms after LT is about two to five times higher than in the general population [19]. Among patients with hepatocellular carcinoma (HCC) or other liver or bile duct malignancy as the primary LT indication, relative mortality from malignancy seems to be particularly high (47 to 51-fold) [20].

The incidence for tumors of the skin seemed to be lower for LT recipients than for recipients of other organ transplants (heart/lung > kidney > liver). A German retrospective observational cohort study revealed that LT recipients tended to have the lowest risk of developing a first and second post-LT non-melanoma skin cancer (NMSC) while being more likely than other organ transplant recipients to receive immunosuppression in monotherapy (32.6%) [21].

However, data assessing the incidence, dynamics, and impact of predictors for subsequent post-transplant NMSC across diverse types of transplanted organs in order to guide medical decision making in clinical practice are limited. The AASLD and the AST recommend that all patients should be seen by a dermatologist after LT to assess cutaneous lesions (at least annually, Table 1) [2].

In a recent analysis of the U.S. Scientific Registry of Transplant Recipients (SRTR) among 89,036 LT recipients focusing on non-skin malignancies, the cancer risk was more than 11 times higher compared with the general population. De novo non-skin malignancies occurred in 4.32% of LT recipients and were most commonly found during follow-up in the second year after LT. Compared with the general population, the cancer risk gradually increased from the first post-LT year, reaching the highest value at 6–10 years follow-up, while cancer risk was decreased during the first 6 months and after more than 16 years [22]. De novo malignancies among LT recipients also tend to have a more aggressive behavior and are associated with higher mortality than in the general population [22,23,24].

In the literature, the extent to which there is an excess risk in the case of colorectal cancer (CRC) still remains controversial [25,26,27]. However, a systematic meta-analysis found that the risk of developing CRC was 2.5 times higher in LT recipients [28]. Recent data from SRTR linked with 15 U.S. population-based cancer registries (1987–2010) revealed an elevated CRC risk in LT recipients with primary sclerosing cholangitis (PSC) and inflammatory bowel disease (IBD) [29].

In this respect, the recommendation for high-risk groups, i.e., patients with PSC, IBD, or other established risk factors, to undergo an annual coloscopy with biopsies is widely accepted (Table 1).

Most literature data on de novo gastric cancer after LT is derived from small case series in Asia [30,31]. In a retrospective German single-center study with 666 patients who underwent LT between 1993 and 2010, the risk for gastric cancer after LT (*n* = 5/666) was not statistically significant increased [32]. In a study with 11,226 LT patients and 75,708 patient years of follow-up, the esophagus was one of the locations of cancer with an increased incidence compared to the general population of France. The authors point out that the French population included a large proportion of patients with alcohol-related liver disease (ALD) [33], which is an important risk factor for increased cancer rates, especially in the upper gastrointestinal tract, oropharyngeal area, and lung [34,35]. However, implementation of an annual screening examination by an upper digestive endoscopy at a single center in France (adherence rate of 36.5%) had no beneficial impact on survival rates at 1 year and at 5 years [36].

The overall risk to develop prostate or breast cancer after LT did not seem to be higher than in the general population [21,25]. In the recent French large population-based analysis with more than 11,000 LT patients, there was no significantly increased incidence in cancers of the ovaries, breast, or prostate [33]. In a population-based study over three decades of Nordic countries (Finland, Sweden, Norway, Denmark) including 4246 LT recipients, standard incidence ratio for prostate cancer was even significantly reduced compared to the general population [37].

According to a meta-analysis, there is a significantly increased incidence of renal cancer in LT recipients, and thus a yearly routine post-transplant ultrasound screen might be beneficial [38].

An Austrian study examined retrospectively whether an extensive surveillance protocol promotes early diagnosis and improved survival in patients with de novo malignancies following LT. After introducing an intensified surveillance protocol (performed annually in all patients regardless of age including chest and abdominal computed tomography (CT) scan, urological/gynaecological evaluation, and dermatological screening), there was a significant improvement for the detection rate of de novo non-skin cancers (increasing from 4.9% to 13%) without any differences between the tumor entities being observed. Malignancies were also diagnosed at earlier stages [39]. For future protocols requiring annual radiological screening, radiation burden must be taken into account [40].

#### 1.1.4. Recurrent Liver Diseases after LT

The recurrence of hepatocellular carcinoma (HCC) is one of the relapsing liver diseases with the highest clinical relevance [3]. The risk of HCC recurrence is given as 8–20% of LT recipients and is usually seen during the first 2 years after LT, with a median survival lower than 1 year [1]. The liver and lungs appear to be the leading sites for recurrence of HCC after LT [41]. According to the German interdisciplinary guidelines on the diagnosis and therapy for HCC, local liver imaging tests should be performed every 3 months within the first postoperative year and every 6 months after 1 year of local tumor clearance for a total of 2 years; in addition, local imaging follow-up should be combined with a CT chest every 12 months in order to detect lung metastases that often occur later [41].

#### 1.1.5. Infectious Diseases and Vaccination

The risk of infection after transplantation changes over time—also depending on the immunosuppressive medication used. In patients who have good graft function and for whom immunosuppression has been tapered to maintenance treatment (usually after 6 months of LT), the same types of community-acquired infections as seen in the general population are generally developed, although at an increased rate [42]. As a study in Germany has shown, immunization rates were too low in solid-organ transplant recipients, with almost 90% of patients being not adequately informed about vaccinations [43].

According to several guidelines and recommendations, annual influenza vaccination is particularly indicated in LT recipients [1,2,3,44,45]. The STIKO (“Standing Committee on Vaccination”) at the German national public health institute (Robert Koch Institute, RKI) recommends sequential vaccination with the 13-valent conjugate vaccine (PCV13), followed by PPSV23 after 6–12 months in organ-transplanted patients. Splenectomized patients should also be immunized against pneumococcus and *Haemophilus influenzae* (type b, Hib) every 3–5 years, and against meningococcal disease (serogroups A, C, W, Y, and B) [45,46,47]. According to the updated guidelines of the American Society of Transplantation Infectious Diseases Community of Practice (AST IDCOP), inactivated vaccines in the post-transplant setting can be administered starting at 3-6 months post-transplant, except the influenza vaccine, which can be given as early as 1 month post-transplant. In contrast, live vaccines are usually not recommended after transplantation. It should be pointed out that immune response to a vaccine will be impacted by the type and amount of immunosuppression after organ transplantation. The vaccination status should be regularly assessed in all transplant recipients [46]. Further detailed information on vaccinations in patients after a solid organ transplant was published in early 2020 [47].

#### 1.1.6. Bone Metabolism and Dental Health

Loss of bone mass has been reported with end-stage liver disease and may accelerate in the first 4–6 months after LT, independently of the pre-LT bone mineral density. However, in patients with normal graft function, bone metabolism also improves, and bone loss decreases in the first 4–12 months from LT. Both the EASL and the AASLD/AST recommend performing bone mineral density (BMD) screening on an annual basis for osteopenic patients in the first 5 months after LT and every 2 to 3 years for patients with normal BMD, subsequently defining the frequency of BMD testing on an individual basis, dependent on the progression and risk factors [1,2].

Oral and dental health may also be affected by immunosuppression in LT recipients. Moreover, in reverse, the immunosuppressed state bears an increased risk of developing systemic infections in cases of neglectful dental care. Compared to the general population, both LT candidates and LT recipients tend to have poorer dental/oral health [48], which can lead to increased risk for oral infections, oral mucosal lesions, and neoplasms in the oral cavity [49]. However, to date, with limited data, there are no generally valid protocols for dental care available for LT recipients.

#### 1.1.7. Psychological Problems after LT

It is obvious that the impact of psychiatric conditions on the morbidity and mortality of solid transplant recipients in the long term is still largely unexplored. A prospective cohort study (*n* = 134) reported a 17% increase in 5-year mortality risk associated with each additional depressive symptom on a scale with 13 items administered early posttransplant [50]. A cross-sectional survey performed among 373 LT recipients revealed that 33.4% of the respondents experienced clinically relevant symptom levels of anxiety (28.7%), depression (16.5%), or posttraumatic stress (PTS) (10%) [51]. For comparison—depression or anxiety rates during comparable time periods are 3 to 10% in the general population and 10 to 40% in individuals with, e.g., arthritis, malignancies, DM, or kidney disease [52]. Symptoms of anxiety and depression were found to be more prevalent in the first 2 years and in the long term after transplantation, and PTS symptoms were more prevalent in the first 5 years after transplant. However, in the long run (>10 years), prevalence rates of depressive/anxiety symptoms were slightly, but not significantly increased [51]. In a cohort of 167 patients transplanted for ALD-related cirrhosis, LT recipients with increasing/persisting depression within the first post-LT year were more than twice as likely to die (all-cause mortality) within the subsequent years. Ten-year survival rates for patients with high (or increasing) depressive symptom levels were 43–46%, compared to 66% among recipients with low depression levels [53]. Interestingly, LT recipients of this cohort who received “evidence-based pharmacotherapy” for early post-LT depression had long-term survival equivalent to that of non-depressed LT recipients [54]. A meta-analysis of 27 studies including 6 studies in LT recipients revealed that depression increased the relative risk of mortality by 65%, while the association with anxiety appeared not to be significant. This effect size is within the range of depression mortality associations described in cohorts with, e.g., lung, heart, or kidney disease or cancers [52].

#### 1.1.8. Immunosuppressive Drug Level Monitoring

The development of effective immunosuppressive therapies contributed significantly to improved graft and patient survival post-transplant, reducing the rate of graft loss from acute and chronic rejection. The mainstay of maintenance immunosuppression is a calcineurin inhibitor (CNI) in Europe and in the USA, with nearly 97% of LT recipients discharged from the hospital on CNIs [2]. In addition, antimetabolites such as mycophenolate mofetil (MMF) are increasingly being used—including to reduce CNI exposure also [1].

A protocol immunosuppression that meets all LT recipients’ needs does not exist and the overall approach to immunosuppression seems to vary widely between transplant centers. A good insight into the heterogeneity of immunosuppressive regimes selected and used following LT at German transplant centers is provided by a review and survey from 2015 [55].

Tailoring immunosuppression includes, for instance, adjustment of the regimen when renal function is impaired, HCC recurrence/extrahepatic malignancy has to be avoided, and if metabolic complications occur. It has been recommended that every patient’s immunosuppressive regimen should be reviewed at least every 6 months and modified as required with the goal of minimizing long-term toxicities [2]. Among the CNIs, tacrolimus is the drug of choice in almost 90% of liver-transplanted patients [1]. However, the narrow therapeutic range of tacrolimus is making therapeutic drug monitoring essential to individualize the tacrolimus dose [56].

#### 1.1.9. Liver Damage, Fibrosis, and Graft Failure after LT

There is still a considerable number of patients developing graft failure after LT, and 10–19% of patients requiring retransplantation. In a recently published review of clinical data and histology from patients who underwent liver retransplantation at King’s College Hospital in London, the proportion of chronic rejection declined, while that of unexplained chronic fibrosing hepatitis increased steadily, representing a second main reason for retransplantation conducted in adults in the most recent era (2002–2014) [57]. Importantly, mild refibrosis and changes of subclinical rejection usually occur with normal or only slightly elevated liver function tests [58]. Rejection can be reliably diagnosed only on the basis of liver histology [2].

A retrospective evaluation of 404 patients who underwent LT between 1998 and 2008 at the university hospital of Mainz in Germany found that even for a subgroup of patients with fast fibrosis progression and recurrent cirrhosis, the protocol biopsy was too late after 1 year from transplantation [59]. A cohort study on the impact of liver biopsies in LT recipients (*n* = 312) showed that the immunosuppressive therapy was adjusted in 41% of the cases after liver biopsy (52% were event-driven liver biopsies due to suspected cellular rejection vs. 14% due to protocol biopsy) [60]. However, non-invasive tests such as vibration-controlled transient elastography (“FibroScan”) are easier to perform, can be regularly repeated, and have the potential to detect early graft fibrosis; some studies even relate increased graft stiffness to adverse prognosis [61,62], and thus the non-invasive form of transient elastography remains the method of choice in clinical routine.

Although not yet finally understood, there is some evidence that the development of donor-specific antibodies (DSAs) in LT recipients should be considered as an independent risk factor for graft loss [63,64,65]. Moreover, in the first year after LT, underimmunosuppression has been associated with increased levels of de novo DSAs [66]. Positive DSAs in combination with subclinical rejection by histology were indicative of more advanced fibrosis, and DSAs can be associated with a molecular pattern resembling T cell-mediated rejection [67].

#### 1.1.10. Immunosuppressive Drug Adherence

Generally, it has been established that post-transplant nonadherence to immunosuppressive therapy is an independent risk factor for poor outcomes. In LT, low adherence during the first 16 to 18 months post-LT has been associated with a higher risk of graft loss [68]. A prospective nation-wide cohort study from Switzerland (*n* = 1505) reported continuously increasing post-transplant medical nonadherence from month 6 to year 3 post-transplant. In addition, pretransplant medication nonadherence was predictive for later immunosuppressive medication nonadherence, indicating the importance of early adherence-supporting interventions [69]. Therefore, it is important to maintain clinical awareness of risk factors of nonadherence, be it at the medication level (e.g., fluctuations in drug levels, development of de novo DSAs, prescription frequency) or be it in terms of patient-related factors (e.g., patient’s mental/social status, adverse effects of medication, history of substance abuse) [44].

However, only a few studies have focused on the problems of improving immunosuppressive medication adherence in LT recipients. In a single center study at the Medical School of Hannover, physicians tended to use observable cues such as sex, language skills, or elevated anxiety and depression scores in order to draw conclusions regarding adherence level. Moreover, the physicians who were asked to estimate their patient’s adherence (1 year after kidney transplantation) tended to underestimate patients’ nonadherence to immunosuppressive medication [70].

#### 1.1.11. Setting of Outpatient Aftercare and Costs

Currently, neither standards regarding training level nor the numbers of staff for the outpatient aftercare setting of LT recipients are defined in Germany. Even at international level, there is hardly any data on which follow-up structures are most likely to improve the long-term outcome of LT recipients. The only available study regarding practice patterns in the management of metabolic complications after LT addressed transplant hepatologists in the USA (*n* = 280) using a postal survey, revealing a great variation with respect to the number of posttransplant clinics, the clinic format, and the number of recipients cared for per week. According to respondents, hepatologists (66%) and primary care physicians (PCPs, 24%) were primarily responsible for the overall care of LT recipients 1 year or more after LT. The majority of hepatologists indicated that ideally PCPs should manage conditions such as hypertension, DM, dyslipidemia, or bone disease, but they also felt that this often was not the case in practice. A majority of those surveyed felt that communication from the PCP to the LT center was inadequate [71].

The difficult resource allocation between the interfaces of the different care sectors can also represent barriers of an optimal intersectional collaboration [72]. Insights into cross-sectoral cost structures of LT in Germany have been limited to monocentric evaluations, and thus conclusions about the effects of different reimbursement models are also not possible [73,74]. However, a German analysis of the main cost drivers revealed the dominating role of costs incurred by inpatient care for the treatment of LT recipients, while post-LT outpatient medical services (2%) and post-LT rehabilitation costs (<1%) were only a small part of the total costs. Seventy-five percent of these follow-up-associated costs were caused by pharmaceuticals, 21% by inpatient services, 4% by outpatient services, and <1% by rehabilitative services [73].

## 2. Materials and Methods

A round table with a group of 12 German transplant hepatologists and liver transplant surgeons was held in September 2019. In order to obtain a first insight of the various follow-up systems across the country, the participating centers agreed to take part in a written survey prior and after the meeting.

The participating LT units represented almost all regions of Germany (12/22) and at least 60% of the total annual volume of livers transplanted in Germany. The authors’ centers answered questions about the current situation, followed by questions about the desirable (“target”) state of post-LT follow-up in a second survey. The questions were standardized, with four of them formulated as completely open-ended (“medical challenges”, “immunosuppressive medication/adherence”, and “reimbursement/structural challenges”). A summary paper based on a review of the literature (PubMed) and personal experience was created to focus on the status quo in the German long-term post-LT management.

## 3. Results

### 3.1. Perceived Risk Factors that Affect the Post-LT Outcome

**Figure 1:**
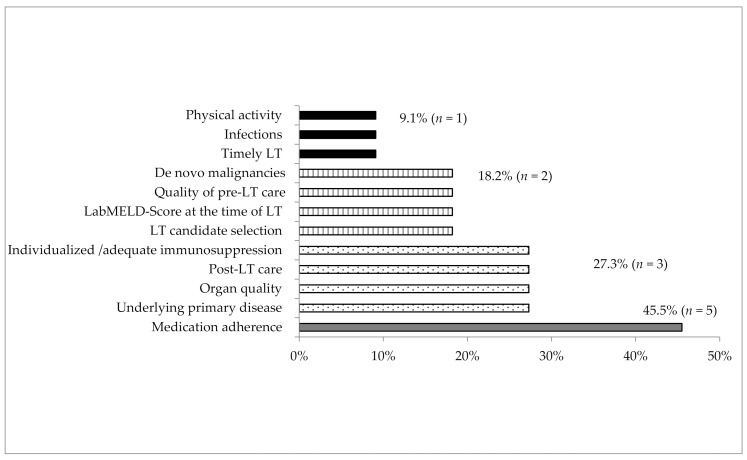
Responses to the open-ended question: “Which factors have the most likely influence on the patient outcome in the post-transplant setting?” (11 centers; multiple answers possible).

When asked an open-ended question about factors that are currently most likely to influence the long-term outcome of their LT recipients, participants most frequently highlighted the factor of “medication adherence” (*n* = 5) next to “the underlying primary disease” (*n* = 3). Other factors included “organ quality” (*n* = 3), “good quality of post-LT care” (*n* = 3), “individualized immunosuppressive management”/“adequate blood level of immunosuppressive drugs” (*n* = 3), “candidate selection” (*n* = 2), “level of labMELD-Score at the time of LT” (*n* = 2), “quality of pre-LT care” (*n* = 2), “development of de novo malignancies”/“malignancy screening in the post-LT setting” (*n* = 2), “timely LT” (*n* = 1), post-LT “infections” (*n* = 1), and post-LT “physical activity” (*n* = 1) (Figure 1).

### 3.2. Evaluation of Metabolic Risk Factors

The majority of the authors’ LT centers surveyed in Germany (*n* = 11) had no standard procedures for screening LT recipients with regard to metabolic and/or cardiovascular risk factors, resulting in different procedures at each center. When asked how often a cardiological check is currently performed, 45.5% of the LT centers said “annually” (*n* = 5), “less frequently than once a year” (*n* = 3), or “not at all” (*n* = 2). An ophthalmological examination of LT recipients to exclude diabetic retinopathy was most frequently sought “annually” (45.5%; *n* = 5) and “less frequently than once a year” (27.2%; *n* = 3). Those who answered “others” (*n* = 2) stated that, in particular, patients with known diabetes and/or hypertension would be seen regularly by the ophthalmologist.

It was mentioned that patients with impaired renal function generally receive nephrological care.

### 3.3. Cancer Screening

**Figure 2:**
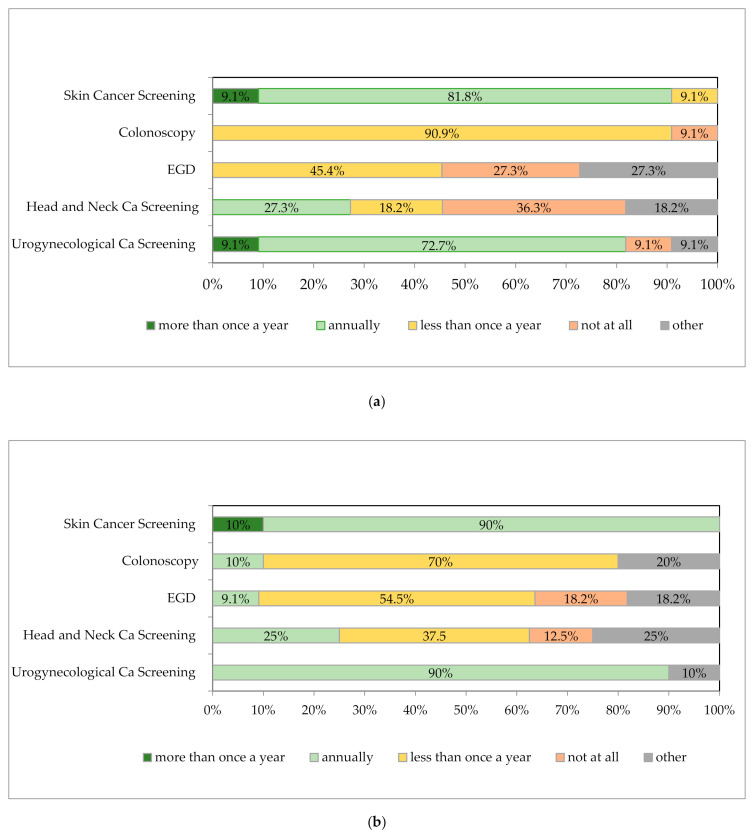
Follow-up procedure at German liver transplantation (LT) centers. (**a**) Cancer screening at current state (*n* = 11). (**b**) Cancer screening at target state (*n* = 10). Abbreviations: EGD = esophagogastroduodenoscopy, Ca = cancer.

The majority of authors’ centers in Germany (81.8%; *n* = 9) stated that they were currently sending their patients to undergo skin cancer screening “annually” (Figure 2a).

In nearly all surveyed LT centers (90.9%; *n* = 10), the LT recipients were currently being sent to colonoscopy screening “every 5 to 10 years”. In contrast, 45.4% of the centers advised their patients to have a check-up esophagogastroduodenoscopy (EGD) performed “less than once a year”, and 27.3% did not aim EGD at all (“not at all”).

The answers of authors’ LT centers regarding their screening procedures for head and neck cancers varied considerably. Only 27.3% (*n* = 3) sent their patients to an annual screening (“annually”), while 36.3% made no general “recommendation” at all (“not at all”, *n* = 4). Two centers (18.2%) stated that they send their patients to screening for head and neck cancers “less than once a year” (Figure 2a).

Although age-specific adjustment is possible, the majority of the authors’ centers surveyed (72.7%; *n* = 8) sought a regular uro-gynecological screening on an annual basis for their LT recipients (“annually”, Figure 2a).

### 3.4. Screening for Liver Diseases after LT by Imaging Modalities

More than half of the centers currently endorsed an at least “annual” ultrasound examination of the liver (54.6%; *n* = 6), with 45.5% (*n* = 5) even explicitly supporting this “more than once a year”. None of the 11 centers surveyed performed “less than once a year” or did without a doppler ultrasound of the liver (“not at all”). In contrast, elastography-based imaging of the liver was performed “less often than once a year” by most centers (63.6%; *n* = 7).

The majority of the responding 11 authors’ centers (72.7%; *n* = 8) stated that they currently scheduled post-LT screening for HCC recurrence “more than once a year”, and 27.3% (*n* = 3) “annually”.

### 3.5. Vaccination Counselling in LT Recipients

The majority of the centers surveyed indicated that they were sending their patients for post-LT vaccination counselling on an annual basis (72.7%; *n* = 8).

### 3.6. Bone Metabolism and Dental Health

A bone mineral density (BMD) screening for LT recipients was usually suggested less frequently than once a year (72.7%; *n* = 8). Currently, over half of the surveyed German LT centers would send their patients to an annual dental check-up (54.5%; *n* = 6).

### 3.7. Psychological and Psychiatric Monitoring

Only a small proportion of the centers surveyed were able to offer regular psychological or psychiatric evaluation. Many centers could not offer their patients to undergo psychological/psychiatric assessment (not at all, 45.4%), particularly due to the lack of implementation options.

### 3.8. Immunosuppressive Drug Level Monitoring

At the surveyed authors’ centers (*n* = 11), immunosuppressive drug levels within the first month after LT were mostly measured “more than once a week” (54.5%), with four LT centers responding that they performed drug level monitoring “less than once a month” (36.4%). If the centers were surveyed about the first 3 months since LT, the majority (45.4%; *n* = 5) preferred a “weekly” interval for the immunosuppressive drug level monitoring. Within the first year after LT, the majority of LT centers performed immunosuppressive drug level monitoring “less than once a month” (54.5%). One year after LT, most of the drug level measurements were performed “less frequently than once a month” (72.7%).

When asked if the immunosuppressive treatment was adjusted at certain time intervals, a majority of 72.7% (*n* = 8) of the centers confirmed that this was the case. In most cases (62.5%; *n* = 5), immunosuppressive medication was adjusted after a time period of 3 months, in one case at 6 months (12.5%). In the remaining cases, the drug adjustment was made continuously or on an “individual” basis. The most common reasons that led to the adjustment of immunosuppression were in the order of frequency, renal impairment (*n* = 11), de novo extrahepatic malignancies (*n* = 10), acute or chronic rejection of the graft/neurotoxicity/HCC before LT, metabolic complications (each *n* = 9), fluctuations in drug levels/diarrhea (each *n* = 8), adherence issues (*n* = 7), HCC after LT (*n* = 6), and development of de novo DSAs (*n* = 5).

### 3.9. Liver Biopsy Monitoring

**Figure 3:**
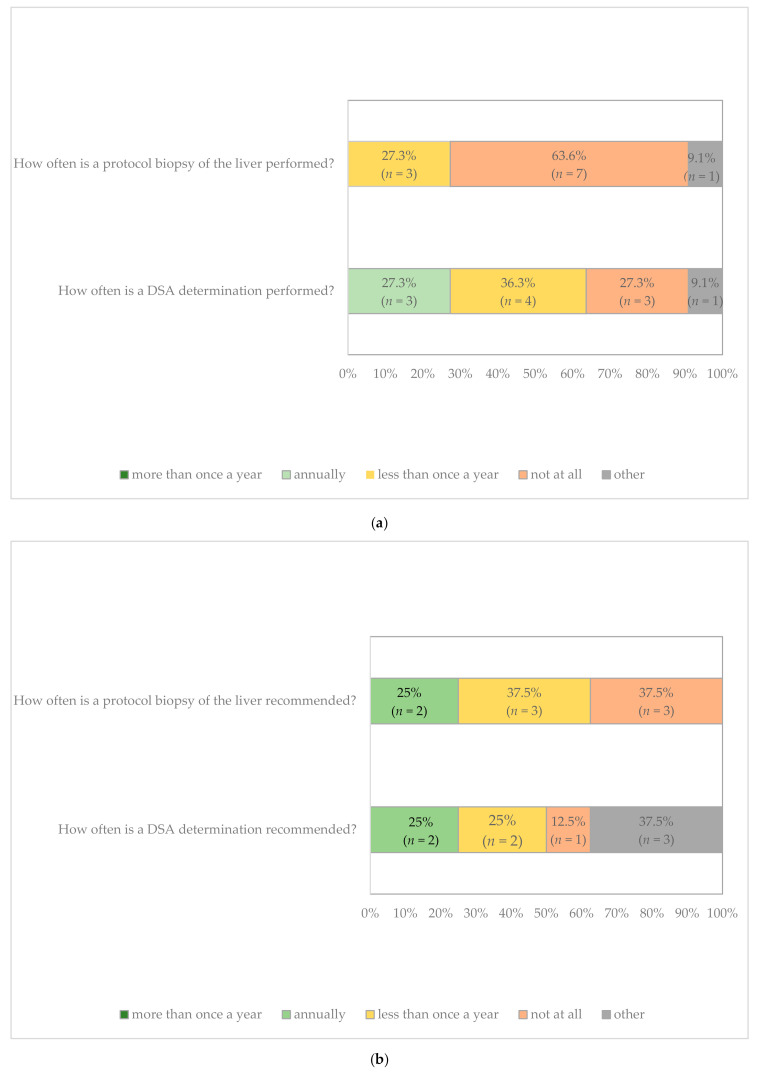
Follow-up procedure at LT centers. (**a**) Current state for protocol biopsy of the liver and DSA (donor-specific antibody) monitoring (*n* = 11). (**b**) Target state for protocol biopsy of the liver and DSA monitoring (*n* = 8).

According to the survey, many centers refrained from suggesting that patients undergo protocol liver biopsies (“not at all”, 63.6%). Three centers performed protocol biopsies (27.3%), but less frequently than once a year (Figure 3a).

### 3.10. Donor-Specific Antibody (DSA) Monitoring

Three of the responding 11 LT centers (27.3%) indicated that they are currently performing an annual DSA evaluation, and four centers (36.4%) reported DSA evaluation less frequently than once a year, while another three centers (27.3%) performed no standard monitoring of DSAs at all (Figure 3a).

### 3.11. Monitoring Immunosuppressive Drug Adherence

All of the responding authors’ LT centers agreed that regular contact with therapeutic drug monitoring is one prerequisite for achieving a good adherence to immunosuppression, followed by “choice of the dosing regimen” (72.7%; *n* = 8); “dosage form” (63.6%; *n* = 7); “medication reminder” such as, e.g., handy alarm, reminder app (45.5%; *n* = 5); or “printed leaflets” (27.3%; *n* = 3).

### 3.12. Setting at LT Centers

Questioned on the actual setting and size of LT outpatient centers, the majority (72.7%) of authors’ centers stated that they were responsible for the aftercare of more than 500 LT recipients. In most outpatient clinics, there is one senior physician who is supported by several assistant doctors (54.5%). In most of the surveyed outpatient clinics, transplant nurse specialists are involved in the outpatient care (72.7%). However, all of the remaining centers (*n* = 3) would appreciate the recruitment of a specialized nurse (Table 2).

At the same time, many of the authors’ LT centers did not feel adequately financially compensated (58.3% of the surveyed LT centers found the current billing modalities satisfactory, but 41.7% did not), with some of the payment practices not allowing for cost-effective work. When asked about the remuneration system, 3 of 12 responding LT centers (25.0%) indicated that billing was performed via an institutional authorization.

Most of the surveyed centers (88.9%) preferred a frequency of follow-up individually tailored to the patient (every 3 months, every 6 months, or annually), depending on the time interval to the LT. Only one center reported that all patients were placed on a follow-up every 3 months.

## 4. Discussion

The present survey results give a first impression of the great variability but also the intersections of the aftercare setting and follow-up systems among LT centers across Germany (current and target state), the sometimes suboptimal staffing in outpatient clinics and lacking definitions of adequate care structures, as well as the planning of an age-appropriate aftercare of LT recipients (e.g., early vs. late aftercare). It is obvious that post-LT care is complex and requires a comprehensive, interdisciplinary approach, especially since the leading post-LT causes of death in the long run are cardiovascular diseases, malignancies, and liver disease recurrences. Even if metabolic risk factors such as dyslipidaemia, obesity, DM, and the development of comorbid CKD usually increase with age, LT recipients alive at 1 year post-LT face a 2.4-fold higher risk of death and a 5.8-fold higher risk of premature death before the age 75 compared to general population [20]. In addition, NAFLD and NASH—both recurrent and de novo—are common after LT and are linked to increased CV disease risk [2]. This underlines the importance of prevention and treatment of pre-existing or new-onset cardiovascular risk factors and recurrent liver diseases, as well as regular cancer screening. Interestingly, the authors’ centers set different priorities (e.g., medication adherence) in the survey, when they were asked with regards to the most important factors influencing long-term survival of LT recipients. Moreover, the majority of the authors’ centers surveyed had no standard procedures for screening LT recipients with regard to metabolic and/or cardiovascular risk factors. After all, almost half of the authors’ centers stated that they send their patients to a cardiological check-up once a year. When the expert panel was asked about their ideal conception in this regard (“target state”), they stated that metabolic syndrome is to be screened on an individualized basis, depending on risk factors (e.g., time from LT, age, underlying primary disease, smoking status, comorbidities). Indeed, those LT recipients with metabolic syndrome were approximately four times more likely to have a CV event [75]. According to population-based data, the CV risk seems to be particularly elevated in patients transplanted for primary biliary cholangitis (PBC), ALD, and hepatitis C (when including HCC) [20]. Even if there are currently no specific recommendations for the prevention of NAFLD in LT recipients, and general interventions similar to those for the normal population apply (avoidance of weight gain, adequate managed DM, and hypertension), intensified treatment of modifiable factors should be aimed to ensure improved long-term survival.

Since de novo malignancies are a major risk factor for decreased long-term survival, cancer surveillance after LT has also high priority, especially in patients with high-risk features. According to the population-based data from the Nordic LT registry, the highest overall standardized incidence ratio was noted in patients transplanted for ALD (especially for lung cancer, pancreatic carcinoma). In patients transplanted for PSC, a fourfold risk for CRC was observed; PSC patients with IBD are also considered high-risk patients [37]. Interestingly, in the Nordic LT registry, a decreasing cancer risk was observed for malignancies that are strongly associated with immunosuppression (in particular for NMSC) occurring within 10 years post-LT from the 1980s [37]. The majority of surveyed centers stated that skin cancer (81.8%) is intended on an annual basis. It was mentioned that the screening costs for skin cancer are only reimbursed every 2 years if a higher frequency is not justified by the dermatologist. However, the vast majority (90%) were in favor of annual screening (Figure 2b, “target state”).

The evidence for other malignancies (e.g., head and neck cancer, gastric and uro-gynecologic cancer) is somewhat less clear. However, in a meta-analysis of 10 studies, standard incidence ratios for head and neck cancer was nearly fourfold higher than in the general population [35]. Some experts confirmed from their clinical experience that patients with a known history of smoking and/or ALD should be regularly screened for head and neck cancer, with the survey results showing no clear direction (Figure 2a,b). However, a standardized survey of smoking status in liver transplant patients is currently not carried out in most of the surveyed LT centers. The reluctance to recommend regular EGD appears to be justified in view of the limited data. In nearly all authors’ centers, LT recipients were currently being sent to colonoscopy screening “every 5 to 10 years”. Here, too, depending on the risk group, a different approach might be worthwhile (e.g., annual screening for patients with PSC and IBD) [1]. Screening for prostate/breast cancer and cervical carcinoma is primarily based on the general medical check-ups for non-LT individuals in Germany and therefore does not reflect any LT-specific data (e.g., increased renal cancer risk).

It was discussed that the frequency of screening for non-skin tumors should be personalized to patients’ individual risk factors, including age and duration of immunosuppressive therapy, as well as other existing risk factors (Table 3). According to experts’ opinion, compared to the general population, usually a higher frequency of check-up examinations is aimed at in long-term adult transplant recipients, although evidence of clinical studies is missing.

In terms of screening for liver diseases, the majority of the authors’ centers currently consider an ultrasound examination at least in annual intervals (54.6%) and/or an annual elastographic examination (63.6%) of the liver to be justified. Even higher rates for annual elastography was indicated for “target state”, while 40.0% (4/10) advocated ultrasound scans once a year (4/10) and 60.0% (6/10) more than once a year. Screening for post-LT HCC recurrence is aimed at for “more than once a year” (72.7%) in most centers, although the guidelines recommend even more frequent screening intervals [41]. The experts emphasized that HCC follow-up phase must be followed by preventive care, as around 40% of recurrences occur more than 2 years later. Of note, ultrasound examination and elastography will continue indefinitely. Two centers stated at “target state” that they would actually like to use the biopsy annually. If the biopsy is performed annually (not the case at our surveyed centers), this applies to the first 5 years.

With regard to vaccination counseling, the majority of surveyed centers followed the national recommendations for immunosuppressed organ transplant patients, although individual adaptations for pediatric patients were mentioned. A BMD measurement was supported by the majority less than once a year, and half of the authors’ centers recommended an annual dental check-up. Of note, BMD screening is covered by German public health insurances only in certain cases (and then not annually).

Almost half of the centers (45.4%; *n* = 5/11) stated they had no option to implement psychological/psychiatric evaluation for their organ transplant patients. However, 55.6% (5/9) would support such an investigation on an annual basis. Since the available evidence suggests a correlation in particular between depression and higher post-LT mortality, which corresponds to other serious chronic conditions such as cancer or DM, the detection of depression should be part of the follow-up care. The most promising is the use of validated assessment tools such as the Beck Depression Inventory-II or Patient Health Questionnaire-9 to detect clinically significant depression [52]. Randomized studies that examine the influence of antidepressant therapies on post-LT survival are lacking. However, there are first data for patients with ALD-related LT that early treatment with antidepressant drugs may lead to a normalization of the depression-related increased mortality [54].

Immunosuppressive drug level monitoring is currently mostly performed more than once a week (54.5%) or less than once a month (36.4%) during the first month after LT, while being performed weekly by 45.5% in the first 3 months after LT. Many centers performed immunosuppressive drug level monitoring less than once a month within the first year after LT (54.5%). When asked about the “target state”, most centers preferred “more than once a week” (54.5%) or “at least weekly” (36.4%) within the first month after LT. More centers wished to perform at least monthly immunosuppressive drug monitoring (36.4%) than currently possible (18.2%). All of the responding author’s LT centers agreed that the regular contact with therapeutic drug monitoring is one prerequisite for achieving a good adherence to immunosuppression. However, the costs of many services thar are provided as part of immunosuppressant therapeutic drug monitoring are often not covered.

Protocol biopsies (every 5 years) are usually recommended for patients transplanted with autoimmune hepatitis; in individual cases, liver biopsies may be required to confirm NAFLD/NASH or in case of HCV recurrence [1,2]. Even if protocol biopsies were not carried out at all, or in two cases less than once a year, two authors’ centers endorsed carrying them out annually (“target state”). This seemed justified by the consideration that currently no reliable markers are available to recognize the histological changes of subclinical rejection or increasing graft fibrosis as well as possible damage from overimmunosuppression. Given the improved safety of the procedure, liver biopsy should therefore be recommended at least in cases of unclear elevation of liver function tests or for minimization of immunosuppressive therapy. It might be appropriate to evaluate the risk of rapid (re)fibrosis after 6 months from LT, including a first protocol biopsy as part of the post-LT aftercare. The majority of surveyed German centers deal with non-invasive examinations of the liver such as ultrasound or transient elastography without limits, but an annual biopsy would be considered for the first 5 years at most. Similar to the protocol biopsy, DSA monitoring was not a regular part of the post-LT aftercare at most centers (Figure 3a,b).

The majority (72.7%) of surveyed LT centers are responsible for the aftercare of more than 500 LT recipients in their outpatient clinics. It was found that the staffing level for doctors and nurses is generally unsatisfactory and represents a significant structural weakness at German LT centers. A very heterogenous spectrum of different reimbursement models was revealed, including, e.g., private health insurance, ambulant specialist care for rare liver diseases (covered by legislation, § 116b SGB V), personal authorization, or medical care at the university outpatient clinics. According to the expert panel, an ideal billing model should cover costs and allow patients access to interdisciplinary care, without significant restrictions regarding the follow-up measures that are considered important for effective post-LT care. However, 41.7% of the surveyed German LT centers did not feel adequately financially compensated.

The majority of LT centers (88.9%) preferred a frequency of follow-up individually tailored to the patient (every 3 months, every 6 months, or annually), depending on the time interval to the LT. Since most German LT centers cover a large geographical area, the desired 3-month intervals can often not be adhered to. Alternatively, immunosuppressive drug levels and laboratory values can be sent in regularly, i.e., every 3 months, for assessment in the transplant center in order to avoid personal contact. It was mentioned that there is no cost reimbursement for such a service of the transplant center and the reaction to suboptimal drug levels could be delayed.

**Figure 4:**
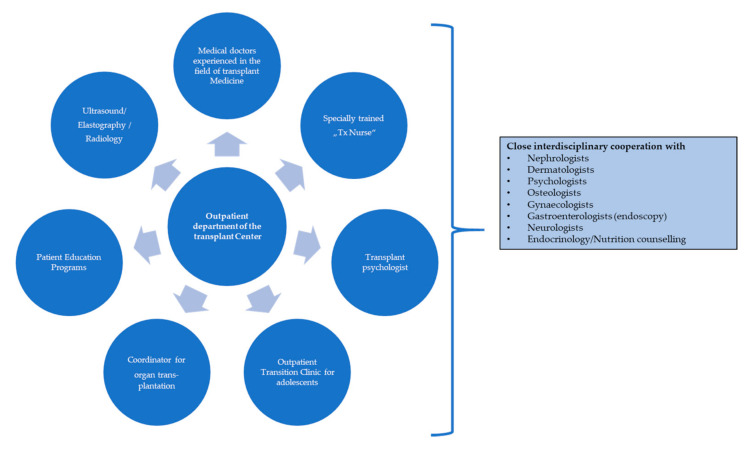
Setting up an interdisciplinary network at transplant outpatient clinics. Tx, transplant.

**Figure 5:**
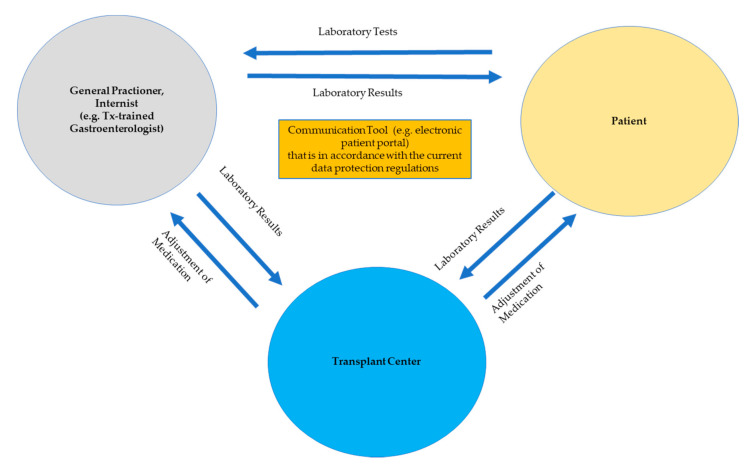
Challenging intersectoral communication and networking.

For optimal coordination and communication between the involved sectors, the transplant center has a leading role, supported by outpatient care through the primary care physicians. An optimized exchange of information between the different health sectors is essential to avoid ambiguity and prevent information loss. Integrating a specially trained nurse who coordinates, implements, and evaluates the care procedures for LT patients could be an important option to overcome the interfaces between the different healthcare sectors. More or less all LT centers have established such networks, which are currently mostly driven by the LT center and are not financially supported by the health insurances or the community. A prototypical example of such a structure is the UKE (University Medical Center Hamburg-Eppendorf) outpatient clinic, which offers a high level of LT aftercare provided by a comprehensive team of, e.g., medical specialists, trained nurses, and transplant psychologists (Figure 4). As the expectations are not always clearly defined within the network of doctors involved in LT aftercare, the exact role expected of each may be indistinct. Ideally, the PCPs control the laboratory values and adjust drug levels, perform the vaccinations, organize the check-ups, and monitor the adherence of the patients (Figure 5). They should be also the first point of contact for infections and should provide social medical support. The LT center should be completely responsible for the immunosuppressive therapy and should also check on all co-medications needed for metabolic syndrome or other comorbidities. In addition, the LT center should be the main contact point if graft function loss/recurrence of primary disease of the liver occurs, if there are unclear symptoms, and for the management of all severe diseases (e.g., post-LT malignancies, severe infections). To reduce costly and time-consuming on-site visits, the use of telemedicine applications such as videoconferences through Skype may be of interest [76]. In the future, the development of an electronic patient portal as an interactive online tool would facilitate communication between the patient, outpatient clinic, and PCP on a consistent basis including the possibility of adjusting the immunosuppression online (Figure 5). However, these technologies must be in line with the complex regulations for data protection.

### Limitations

We acknowledge that the survey only covers a sample of LT centers in Germany, and thus they cannot serve as a basis for definitive results. No data were obtained on the actual prevalence of the corresponding medical complications or the control of these complications. Despite these limitations, the descriptive information should be suitable to give first insights into German post-LT care structures, allowing for discussion of future research and studies needed in this field.

## 5. Conclusions

Our survey-based analysis of post-LT aftercare structures highlighted challenges and heterogeneous approaches at German liver transplant centers. Additional research is needed in order to identify and manage the most relevant risk factors that are responsible for a shortened long-term survival of LT recipients. The development of appropriate follow-up algorithms for different medical post-LT surveillance aspects and the adoption of available guidelines (e.g., referring to DM, NAFLD/NASH, or CV disease) for the LT recipient population might be helpful. Future research is also needed to clarify suitable follow-up intervals (e.g., organ system-related). Currently, there is also a high unmet need to recognize and improve critical issues at the interfaces of different care sectors. In the future, an analysis of the different forms of compensation of healthcare providers and the development of effective approaches to overcome insufficient reimbursement of services at the interfaces between cross-sectoral aftercare structures might be useful. Finally, prospective observational cohort studies will be needed to prove that adjustment of coordination of aftercare structures and specific post-LT surveillance strategies will lead to improvements in long-term graft and patient outcomes.

## Figures and Tables

**Table 1:** Proposals for screening: selected recommendations from the literature [1,2,3,14,18,19].

Dermatologic assessment	Annually [3] At least an annual evaluation by a dermatologist 5 years or more after LT [2] Annual skin examination in “low-risk patients”, every 3 months in high-risk patients (older age, at LT, skin phototype II-III, cyclosporine-based IS) [14]
Other examinations	Annual gynecological/urological assessment, fecal occult blood test [3] Ear, nose, throat examinations in patients with LT for ALD, positive smoking history (annually?) [18]
Mammography	Every 2 years in female recipients older than 50 years [3]
Monitoring of CV risk factors	Cardiac stress test and 24 h blood pressure monitoring (annually, [3]) Fasting lipid profile (annually, [3])
Liver sonography Liver elastography (TE) Liver protocol biopsy	Annually [3] “No evidence that routine protocol biopsy in PBC LT recipients will improve outcomes” [2] Liver biopsy and TE as part of regular assessment of graft damage in patients with recurrent HCV after LT [1]
Colonoscopy	Annual colonoscopy in patients with IBD, every 5 years in patients >55 years [3] Colonoscopy 2 years post LT, annual colonoscopy in patients with PSC+IBD [1,19], consider baseline colonoscopy in patients <50 years, perform baseline colonoscopy in patients ≥50 years [18]
Ultrasound (abdominal) CT MRI and/or MRCP	Annual abdominal ultrasound (renal cancer) [18] Annual thoracic CT/abdominal CT or MRI in patients transplanted with HCC (for up to 5 years [3]) Annual thoracic CT in active smokers [18] MRI and/or MRCP in patients transplanted for PSC (annually?) [3]
BMD testing by DXR	Yearly for patients with pre-existing osteoporosis/osteopenia and every 2 to 3 years in patients with normal BMD [1] Yearly in the first 5 years post LT for osteopenic patients and every 2 to 3 years for patients with normal BMD; thereafter screening depending on the progression of BMD and on risk factors [2] Every 5 years [3]
Vaccination counselling	“Appropriate advice” regarding vaccination after LT [2]

ALD, alcohol-related liver disease; BMD, bone mineral density CV, cardiovascular; DXR, digital X-ray radiogrammetry; HCC, hepatocellular carcinoma; HCV, hepatitis C virus; IBD, inflammatory bowel disease; IS, immunosuppression; LT, liver transplantation; MRCP, magnetic resonance cholangio-pancreatography; MRI, magnetic resonance imaging; PBC, primary biliary cholangitis; PSC, primary sclerosing cholangitis; TE, transient elastography.

**Table 2:** Characterization of the outpatient center setting at liver transplant units in Germany (*n* = 11).

How many patients are embedded into regular follow-up care in your outpatient clinic?		
0–100 patients	0%	(*n* = 0)
101–500 patients	27.3%	(*n* = 3)
500 patients	72.7%	(*n* = 8)
Who is responsible for the medical care of outpatients?		
1 senior doctor, 1 assistant doctor	18.2%	(*n* = 2)
1 senior doctor, assistant doctors in rotating positions	54.5%	(*n* = 6)
1 specialist doctor alone	9.1%	(*n* = 1)
1 senior doctor alone	9.1%	(*n* = 1)
Other * * 1 senior doctor (renal transplant), 1 senior doctor (liver transplant), 1 rotating assistant (surgery), 1 rotating assistant (internal medicine)	9.1%	(*n* = 1)
Is a transplant nurse specialist part of your outpatient care team?		
Yes	72.7%	(*n* = 8)
No	18.2%	(*n* = 2)
Other * * partially, 1 nurse is experienced transplant nurse, a majority are medical assistants (MFA)	9.1%	(*n* = 1)
Would you support the establishment of such a body (transplant nurse specialist)?		
No	0%	*(n* = 0)
Yes	100%	*(n* = 3)

MFA, medizinische Fachangestellte (medical assistant).

**Table 3:** Statistically relevant risk factors for de novo cancer development, based on current literature [18,40,44].

Head and neck cancers	Smoking, LT for ALD
Kaposis‘s sarcoma	Infection with HHV-8, increased intensity of IS, (noteworthy that virus-related developing of Kaposi’s sarcoma seems to occur mainly in Mediterranean populations)
Lung Cancers	Smoking, LT for ALD, HCC
NMSC	Smoking, LT for ALD, age >40 years, male, red hair, brown eyes, PSC, cyclosporine, skin type, sun exposure
Esophageal/gastric cancers	LT for ALD, Barrett‘s esophagus, asian ethnicity
Colorectal cancer	PSC, IBD, pre-LT precursor lesions, obesity/metabolic syndrome (?)
Pancreatic cancer	LT for ALD, PSC
PTLD	Age >50 years, HCV, LT for ALD, use of anti-lymphocyte antibodies

HHV, human herpesvirus; NMSC, non-melanoma skin cancer; PTLD, post-transplant lymphoproliferative disorder.
